# Association between dietary intake and seasonal variations in postmenopausal women

**DOI:** 10.1186/2251-6581-13-52

**Published:** 2014-04-25

**Authors:** Asieh Mansour, Zeinab Ahadi, Mostafa Qorbani, Saeed Hosseini

**Affiliations:** 1 Endocrinology and Metabolism Research Center, Endocrinology and Metabolism Clinical Sciences Institute, Tehran University of Medical Sciences, Tehran, Iran; 2 Chronic Diseases Research Center, Endocrinology and Metabolism Population Sciences Institute, Tehran University of Medical Sciences, Tehran, Iran; 3 Department of Public Health, Alborz University of Medical Sciences, Karaj, Iran; 4 Department of Epidemiology and biostatistics, Iran University of Medical Sciences, Tehran, Iran; 5 Obesity and Eating Habits Research Center, Endocrinology and Metabolism Molecular -Cellular Sciences Institute, Tehran University of Medical Sciences, Tehran, Iran

**Keywords:** Postmenopausal women, Nutrient intake, Diet, Seasonal variation

## Abstract

**Background:**

Evidence supports that increasing number of postmenopausal women are suffering from one or more chronic diseases. Dietary patterns have a pivotal role in maintaining human health. The aim of this study was to characterize the nutrients and energy intake in postmenopausal women, with the special focus on seasonal variation effect in their food intake.

**Methods:**

The study population consisted of 30 postmenopausal women referred to Dr. Shariati Hospital, Tehran (Iran). Socio-demographic characteristics and BMI were registered. Dietary assessment was performed by a 3 day food records in each season through one year, allowing the estimation of energy, protein, carbohydrate, total fat, monounsaturated fatty acid (MUFA), polyunsaturated fatty acids (PUFA) and saturated fatty acids (SFA) intake. The mean of nutrient intake in each season was adjusted for energy intake. The effect of season on energy and nutrients intake was assessed based on the General linear model (GLM).

**Results:**

The mean of daily intake of vitamin C, B, B2, B12, iron, zinc, phosphorus and chromium was significantly higher than Recommended Dietary Allowance (RDAs ) (p < 0.05). The mean of vitamin D, E, B6, B5, folate, calcium, magnesium, potassium and selenium consumption was significantly less than RDAs (p < 0.05). All the participants meet the goal for vitamins A, K and B3 from food. The mean of energy intake was not different between seasons. However, the mean intake of fat, vitamin C, vitamin K and folate was significantly different between seasons.

**Conclusion:**

These findings highlight some nutrients deficiency in postmenopausal women and therefore suggest nutritional education with emphasis on seasonal variation effect.

## Introduction

Menopause is a normal life change in women, characterized by cessation of their reproductive cycle that occurs during late 40s or early 50s [1]. Evidences support that increasing number of women are suffering from one or more chronic disease associated risk factors following ending of their menstrual cycles [2]. Diet and food intake have a pivotal role in maintaining human health. Unhealthy diet, obesity and nutritional deficiencies may lead to various disorders [3]. A better understanding of the factors that contribute to chronic diseases including their habitual nutrient intake is needed in order to modify the diet which can help to prevent or treat the diseases.

In Tehran lipid and glucose study (TLGS), a total of 96 menopausal women were compared with premenopausal for detecting nutritional intake with Estimated Average Requirements (EAR) and The Dietary Reference Intake (DRI) recommendations. The author concluded that in both groups, the mean intake of magnesium, copper, zinc, pyridoxine, calcium and vitamin D was lower than recommendations [2]. Nemati et al. reported that in postmenopausal women, the mean of folate, vitamin B2, vitamin B6, calcium, zinc, selenium and energy intake was less than dietary reference intake [4].

There is some evidences that season could affect the health conditions [5,6]. Some studies showed that season should be regarded as an important risk factor in the incidence of malnutrition and other disorders [7]. The identification of seasonal differences in the nutritional status may advocate the implementation of specific season-related strategy to improve the health of a population [6].

The overall aim of this study was to evaluate the nutrients status in postmenopausal women and also to assess the effects of seasonal changes on nutrient intake of this group of women. Based on our knowledge no study has evaluated the effects of seasonal changes in energy and nutrient intake in our country, despite the existence of four distinct seasons in a year in Iran. The effect of seasonal variation was measured by food record.

## Materials and methods

In a cross - sectional study, 30 postmenopausal women referred to Dr. Shariati Hospital, Tehran, were selected by simple random sampling method and followed for one year. Exclusion criteria were history of drug addiction; body mass index (BMI) ≥35 kg/m2; recent insulin therapy or uncontrolled diabetes; history of cancer or autoimmune, liver or renal disease; a restrictive diet or any change in dietary patterns. Their demographic and clinical details were recorded in a questionnaire. Detailed dietary information was assessed by a monthly (2 weekdays and 1 weekend) Food Record in each season; throughout the year. A small scale was given to subjects and they were instructed how to use it. They were requested to use scale in addition to household cups for a more accurate recording of food intake. Data was collected by a well trained telephone interviewer each month. Nutritionist IV software (The Hearst Corporation 1994) was used to analyze the nutrient and energy intake of the participants.

Height was measured without shoes to the nearest 0.5 cm using a Seca stadiometer. Weight was measured with minimal clothing and without shoes to the nearest 0.1 kg using Seca scale. Body Mass Index (BMI, in kg m^−2^) was calculated from weight (in kg) divided by square of height (in m). Food portion were measured using Balance Hand Scale. This handy pocket sized scale accurately measures small objects with weights up 100 grams.

The medical ethics committee of Tehran University of Medical Sciences approved this project and all the subjects gave informed written consent.

The data were analyzed using the SPSS version 16 for Windows (SPSS Inc., Chicago, IL). The statistical comparison of nutrient intake with RDA was performed with one sample *t*-test. The means of nutrient intake were adjusted for energy by computing the General linear model (GLM) with the nutrient intake as the dependent variable and the energy intake as the independent variable. The level of significance was considered at p < 0.05.

## Results

A total of 30 postmenopausal women were participated. The mean age of these women was 55 ± 1.2 years. The mean experience time of first menstrual was at age 13.7 ± 1.5 years and menopause at 44.7 ± 12.6 years. The mean of weight and BMI were 71.5 ± 9.4 kg and 27.5 ± 3.9 kg/m^2^ respectively. The mean of daily energy, carbohydrate, protein and fat intake was 1535.3 ± 537.2 kcal, 206.3 ± 71.2 g, 60.7 ± 26.2 g and 54.6 ± 30.03 g, respectively. Overall intakes of calcium/vitamin D supplement, vitamin D supplement and omega-3 supplement were 67.9%, 38.5% and 37% respectively.

The results showed that the mean of daily intake of vitamin C, B1, B2, B12, iron, zinc, phosphorus and chromium were significantly higher than RDAs recommendations (p < 0.05). The mean daily consumption of vitamin D, E, B6, B5, folate, calcium, magnesium, potassium and selenium were significantly less than RDAs (p < 0.05). All of participants meet the goal for vitamin A, K and B3 from food (Table 1).

**Table 1:** The mean daily intakes of nutrients in postmenopausal women in comparison with RDA

**Nutrient**	Mean intake*	**Reference intake**^**¥**^	**P.value†**
Vitamin A(μg)	738.8 ± 6.2	700	0.05
Vitamin E(mg)	4.9 ± 5.7	15	0<001
Vitamin D(μg)	5.3 ± 5.3	15	0.000
Vitamin K(μg)	129.2 ± 98.2	90	0.2
Thiamine(mg)	1.2±0.4	1.1	0.000
Riboflavin(mg)	1.3±0.5	1.1	0.000
Niacin(mg)	14.1 ± 7.07	14	0.7
B6(mg)	1.2 ± 0.8	1.5	0.000
B5(mg)	4.3 ± 1.9	5	0.000
B12(μg)	3.1 ± 4.7	2.4	0.003
Vitamin C(mg)	97.1 ± 6.05	75	0.000
Biotin(μg)	9.4 ± 7.6	30	0.000
Iron(mg)	10.7 ± 5.1	8	0.000
Folate(μg)	288 ± 143.4	400	0.000
Zinc(mg)	8.5 ± 4.3	8	0.02
Magnesium(mg)	242.4 ± 115.5	320	0.000
Chromium(μg)	25 ± 0.6	20	0.000
Selenium(μg)	40 ± 1.01	55	0.000
Copper(mg)	1055 ± 22.13	900	0.000
Calcium(mg)	640.3 ± 357.2	1200	0.000
Potassium(mg)	2443.9 ± 1016.7	4700	0.000
Phosphorus(mg)	937.1 ± 446.5	700	0.000
Total fiber(g)	15.3 ± 12.7	21	0.000
Omega-3(g)	2.1 ± 20.2	1.1	0.3

*Mean ± Sd.

The level of significance was considered at p < 0.05.

†One sample *t*-test.

¥http://www.nap.edu.

Macronutrient and energy intake differences between seasons are explained in Table 2. The results were adjusted for energy. The mean of energy intake was not different between the seasons. Among the macronutrients, only the mean of the fat intake was significantly different between spring and autumn compared with other seasons; higher intake was observed in spring and lower intake in autumn.

**Table 2:** Comparison of mean differences energy and nutrient between seasons

Nutrient intake*	**Spring**						**Summer**				**Autumn**		**Season**
	**Summer**		**Autumn**		**Winter**		**Autumn**		**Winter**		**Winter**		
	***Mean difference***	***P-value†***	***Mean difference***	***P-value†***	***Mean difference***	***P-value†***	***Mean difference***	***P-value†***	***Mean difference***	***P-value†***	***Mean difference***	***P-value†***	***P-value†***
Energy(kcal)	26.8	0.7	98.3	0.2	120.5	0.1	71.5	0.3	93.7	0.2	22.2	0.7	0.4
Protein(g)	0.9	0.7	-0.9	0.7	-4	0.1	-1.8	0.4	-5	0.05	-3.1	0.2	0.2
Carbohydrate(g)	-11.2	0.06	-15.2	0.014	-4.7	0.4	-4	0.5	6.4	0.2	10.4	0.08	0.06
Fat(g)	4.6	0.06	7.2	0.005	3.5	0.1	2.6	0.2	-1	0.6	-3.7	0.1	0.04
Cholesterol(mg)	-11.9	0.5	8.3	0.6	-9.4	0.6	20.2	0.3	2.4	0.9	-17.8	0.3	0.7
MUFA(mg)	0.8	0.4	2.7	0.03	0.1	0.8	1.8	0.1	-0.7	0.5	-2.5	0.04	0.1
PUFA(mg)	4	0.005	3	0.03	2.8	0.04	-0.9	0.4	-1.2	0.3	-0.2	0.8	0.03
SFA(mg)	-0.1	0.8	0.6	0.5	1	0.2	0.7	0.4	1.2	0.1	0.4	0.1	0.5
Total fiber(g)	2.8	0.1	1.7	0.3	-0.1	0.9	-1.1	1.8	-3	0.08	-1.9	0.2	0.2
Vitamin A(μg)	77.1	0.5	113.7	0.3	-23.6	0.8	36.5	0.7	-100.8	0.3	-137.4	0.2	0.5
Vitamin E(mg)	1	0.2	1.4	0.08	1.1	0.1	0.4	0.6	0.07	0.9	-0.3	0.6	0.3
Vitamin D(μg)	-0.2	0.7	0.08	0.9	-0.3	0.6	0.3	0.6	-0.04	0.9	-0.3	0.6	0.9
Vitamin K(μg)	70.6	0.000	70.7	0<001	62.2	0.001	0.09	0.9	-8.4	0.6	-8.5	0.6	0.000
Vitamin B12(μg)	1	0.1	0.9	0.2	0.4	0.5	-0.1	0.8	-0.6	0.3	-0.5	0.4	0.4
Vitamin C(mg)	41.5	0.000	1.3	0.9	-21.1	0.055	-40.1	0.000	-62.6	0.000	-22.4	0.04	0.000
Iron(mg)	1.1	0.03	0.6	0.2	0.9	0.08	-0.5	0.3	-0.2	0.6	0.2	0.6	0.1
Folate(μg)	78.6	0.000	62	0.001	34.4	0.05	-16.6	0.3	-44.1	0.01	-27.5	0.1	0.000
Zinc(mg)	0.7	0.1	0.2	0.6	0.2	0.5	-0.4	0.3	-0.4	0.3	0.009	0.9	0.4
Calcium(mg)	16.7	0.7	-46.1	0.3	19.1	0.7	-62.8	0.2	2.4	0.9	65.3	0.2	0.5
Magnesium(mg)	35.1	0.016	12.6	0.3	13.9	0.3	-22.5	0.1	-21.2	0.1	1.3	0.9	0.1
Omega-3(g)	4.8	0.1	4.8	0.1	1.9	0.5	-0.03	0.9	-2.8	0.3	-2.8	0.3	0.3

The level of significance was considered at p < 0.05.

†Analysis of covariance (ANCOVA).

*Analyses were adjusted for energy intake except energy.

Regarding the monounsaturated fatty acid (MUFA), polyunsaturated fatty acids (PUFA) and saturated fatty acids (SFA) intake, the only season difference was presented for PUFA. Mean consumption of PUFA was increased significantly in spring and decreased in summer.

Table 2 also reports the intake of micronutrients based on the season; Significant differences were observed in vitamin C, vitamin K and folate intake between seasons, lower intakes of vitamin C and higher intake of vitamin K and folate were in summer, spring and autumn, respectively. However, no significant differences in another nutrients intake were noticed.

## Discussion

The study had two main objectives; first: to evaluate the nutrients and energy intake of postmenopausal women and second to evaluate the effect of seasonal variation on daily consumption of micronutrients, macronutrients and energy using food record. Mean intake of vitamin A, vitamin K, niacin was reached the recommended intake of these nutrient elements in our population. other studies have reported similar result in respect to niacin intake [4,8]. The mean intake of calcium and vitamin D in our population was lower than dietary reference recommendation. In a study by Nemati et al. with 924 postmenopausal women from different areas of Ardabil province in Iran, remarkably lower daily intake of calcium compared to world reference was observed. Additionally, Chee et al. reported that women consume less than 800 mg calcium per day [9]. Based on the evidences, menopause is associated with a reduction of estrogen release in women, resulting in decreased bone mineral density and increased risk of osteoporosis [10]. Bone density is associated not only with menopause, but also with other factors including the nutrients such as calcium and vitamin D [11]. It is of interest that some studies showed that low dietary calcium intake (< 700 mg/day) is associated with an increased risk of ischemic heart disease death in postmenopausal women [12]. Several recent publications found a significant association between higher rate of cardiovascular disease and also cognitive impairment in the postmenopausal women and low vitamin D levels [12,13]. The mean of daily folate, vitamin B5 and B6 intake in our subjects was lower than RDA recommendations. Low intake of vitamin B6 was also showed in a recent study performed in postmenopausal women [4]. Deficiencies in vitamin B6 and folate can result in increased levels of homocysteine which latter have been regarded as a risk factor for cardiovascular disease, cerebrovascular disease and Alzheimer’s disease (AD) [14]. Hyperhomocysteinemia is also associated with a diminished bone mineral density and an increased fracture risk [15,16]. The lower level of selenium (an antioxidant mineral) is associated with an increased risk of coronary heart disease, cognitive impairment, and cancers [17-19]. In our study the mean intake of selenium was lower than RDA which is in accordance with findings of other studies [4]. In the present study, the daily magnesium intake was insufficient which is similar to result obtained by Mirmiran et al. [2]. Interestingly, in study of 3,713 postmenopausal women conducted by Chacko et al. dietary magnesium intake was inversely correlated with levels of certain markers of systemic inflammation and endothelial dysfunction [20]. In our study, vitamin E and potassium intake were less than RDA. In a prospective cohort study of postmenopausal women, Zhu et al. reported that high dietary potassium intake may be positively associated with better bone density, suggesting that high intake of this mineral may reduce the osteoporosis incidence [21]. Some observational data support a beneficial effect of vitamin E on incidence of cardiovascular disease, cancer, AD and also hot flashes [22-24].

As a second objective, we evaluated the effect of seasonal variation in daily consumption of micronutrients, macronutrients and also energy using the food record form. The effects of season on dietary intake may depend on the population group, socioeconomic condition, and the weather differences. In general, season effects can be apportioned to differences in food items rather than the intake of nutrients [25]. We should mention that even in the present study we did not evaluate the food items, but we found a nutrient difference between seasons. In our study, the total energy intake didn’t differ between seasons; however, the total fat intake was significantly different between spring and autumn. Fat intake was lower in autumn but increased in spring. In contrast in a study conducted by Rassato et al. in Brazil, the healthy adults consumed less fat in summer [7]. In shanghai women health study (SWHS) with 74,958 individuals, Fowke et al. observed an increase in fat intake in winter [26]. Regarding the types of consumed dietary fats, the authors found that PUFA intake was significantly higher among the participants in spring and lower in summer when compared to other seasons. In our study we found no differences between protein and carbohydrate and energy intake in different seasons. In contrast to our findings, in a study performed on 27 women, the authors found an increased energy intake in winter in compression to summer [27]. Similar results were observed in Spain, though this difference was only observed among men subjects [28]. And also a few studies reported that carbohydrate and protein intakes were varied in seasons [7,26,28].

The seasonal differences may be attributed to the changes in food eating pattern rather than nutrients [25], however, in this study we considered the nutrients and energy intake instead of food pattern. The food pattern effects of seasonal variations require further investigation, particularly in countries with four clearly defined seasons during the year.

Diet assessment methods have limitations that could result in misunderstanding the relationship between diet and chronic disorders. These limitations include sex, body size, education as well as seasonal variation effects [26]. The current study suggests that variation in the nutrients intake in different seasons exists, and that variation could affect the nutritional assessment. We assume that dietary evaluation per each season is needed to be done in order to elevate the accuracy of the results.

One of the strengths of the study was using of food record instead of food recall for nutritional assessment. We used a handy scale in order to have a more accurate determination of dietary intake by our subjects. In fact this method doesn’t rely on subject recall and may be able to reflect the precise food intake. Furthermore, in our study, we administered 12 food records per month with one weekend day each season over one year period. The limitation of study was the small sample size.

## Conclusion

We demonstrated the inadequate intake of some nutrients in postmenopausal women. We also demonstrated that seasonal changes may be a source of variation in dietary intake. We suggest that to promote the nutritional knowledge in this group of women, the nutritional education with emphasis on seasonal variation effect should be considered.

## Competing interests

The authors declare that they have no competing interests.

## Authors’ contributions

All authors read and approved the final manuscript.
